# Supplementary material for: Identification of photoperiod-induced specific miRNAs in the adrenal glands of Sunite sheep (Ovis aries)
Source: Front Vet Sci. 2022 Jul 22;9:888207. doi: 10.3389/fvets.2022.888207 (PMC9354845; doi:10.3389/fvets.2022.888207)
Supplement: Supplementary file 1 [file Data_Sheet_1.ZIP › Supplementary file/miRDeep2 file.zip!/miRDeep2 file/Novel_Structure/LP42A1/Novel_616.pdf]

Provisional ID	:	NC_019471.2_22844
Score total	:	3.6
Score for star read(s)	:	3.9
Score for read counts	:	-3.9
Score for mfe	:	2.1
Score for randfold	:	1.6
Score for cons. seed	:	
Total read count	:	4
Mature read count	:	3
Loop read count	:	0
Star read count	:	1

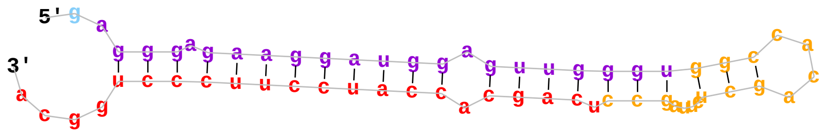

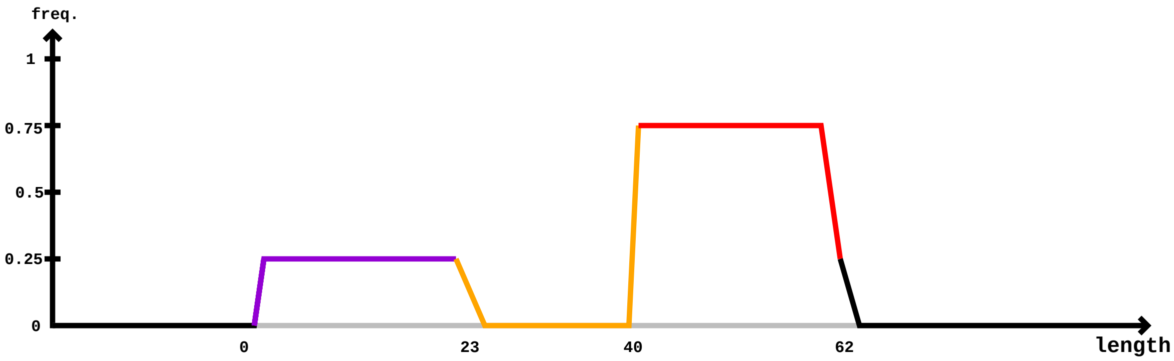

Star Mature

```
5'- agccacuggggucagcagugagggagaaggauggaguugggu ggccacagcucuuagccucagcaccauccuucccuggcaccacugccccuucuugagccucaguuuguac   -3'    obs
    agccacuggggucagcagugagggagaaggauggaguugggu ggccacagcucuuagccucagcaccauccuucccuggcaccacugccccuucuugagccucaguuuguac          exp
    ....((((((((((((((((.(((.(((((((((.((((((((((....)))....))).)))).))))))))))))).....)))))).......))).)))))))......   reads   mm   sample
    ....................agggagaaggauggaguugggu......................................................................   1       0    seq
    ...........................................................ucagcaccauccuucccugg.................................   1       0    seq
    ...........................................................ucagcaccauccuucccuggc................................   1       0    seq
    ...........................................................ucagcaccauccuucccuggca...............................   1       0    seq
```
